# Ethnobotanical knowledge in rural communities of Cordoba (Argentina): the importance of cultural and biogeographical factors

**DOI:** 10.1186/1746-4269-5-40

**Published:** 2009-12-15

**Authors:** Bárbara Arias Toledo, Leonardo Galetto, Sonia Colantonio

**Affiliations:** 1 Cát. Antropología, Facultad de Ciencias Exactas, Físicas y Naturales, Universidad Nacional de Córdoba, Av. Vélez Sarsfield 299 - X5000JJC - Córdoba, Argentina; 2 IMBiV (Instituto Multidisciplinario de Biología Vegetal), Universidad Nacional de Córdoba, Av. Vélez Sarsfieldo 1611 - 5000 - Córdoba, Argentina

## Abstract

**Background:**

The possibility to better understand the relationships within the men, the nature and their culture has extreme importance because allows the characterisation of social systems through their particular environmental perception, and provides useful tools for the development of conservation policies.

**Methods:**

The present study was planned to disentangle environmental and cultural factors that are influencing the perception, knowledge and uses of edible and medicinal plants in rural communities of Cordoba (Argentina). Interviews an participant observation were conducted in nine rural communities located in three different biogeographical areas. Data about knowledge of medicinal and edible plants and sociocultural variables were obtained. Data were analysed by Principal Components Analysis (PCA).

**Results:**

The analysis of data confirmed that medicinal species are widely used whereas the knowledge on edible plants is eroding. The PCA showed four groups of communities, defined by several particular combinations of sociocultural and/or natural variables.

**Conclusion:**

This comprehensive approach suggests that in general terms the cultural environment has a stronger influence than the natural environment on the use of medicinal and edible plants in rural communities of Cordoba (Argentina).

## Introduction

The consumption, management and valuation of wild plants are central aspects of the traditional knowledge in many human populations. Among the potential uses of plants, those related to medicine and foods have central importance because they are essential to human survival. Thus, plant gathering and the diffusion and conservation of knowledge within the community are traditional practices that have contributed to the subsistence of many cultures [1,2]. Farming populations consume leaves and buds in spring when there is no production in gardens [3], fruits of the forest in summer [4], or some of the exotic weeds introduced jointly with the crop fields [5].

The utilization of medicinal plants is still more disseminated around the world [e.g. [6-9]]. In almost all societies, the "scientific-western" medical system coexists with several traditional systems. These traditional medical systems are generally based on the use of natural and local products, which are commonly related to the people's perspective on the world and life [10]. Thus, people's perceptions about the environmental availability of resources would depend on the culture of each society. When some persons describe the use of a plant, they are expressing - in an explicit or implicit way - concepts and categories of their particular culture [11]. Socioeconomic level, age, gender and profession, are some of the variables that may influence the distribution of the knowledge about plant uses within a population [11]. A heterogeneous pattern on plant knowledge within populations of different cultures has been reported elsewhere [[12-15]; etc], as well as for populations from Argentina [16].

Additionally, it is reasonable to expect that the use of vegetal resources also depends on environmental diversity and availability. For example, studies conducted in Argentinean Patagonia have shown that the consumption of edible wild plants among the Mapuches follow a pattern according to the ecological conditions of the gathering environments [17], as their knowledge among teachers [18]. A similar pattern was found in southern Ecuador, where the consumption of edible plants showed large variations thought the region, mainly related to altitude and climate [19]. In this sense, Cordoba province is especially interesting because it shows varied landscapes with distinct biogeographical regions, with different availability of plants.

In summary, perception about the utility of plants must be understood through a dynamic and complex system integrated by people using plants, their culture, and the natural environment from they can obtain plants. That is to say, it would be interesting to disentangle this heterogeneity on the uses of vegetal resources considering both the perception and the knowledge that people have of their environment. The possibility to better understand the relationships among the people, their culture and the environment has central importance because it allows the characterization of social systems through their particular environmental perception, and provides useful tools for the development of conservation policies.

Curiously, this conceptual view that combines data on differential biogeographical availability of plant resources and cultural perception and knowledge of their uses has been scarcely developed in ethnobiological researches. The present study was planned to (a) describe the knowledge and use of edible and medicinal plants in nine rural communities located in three biogeographical regions of Córdoba province (Argentina);(b) disentangle which cultural and environmental factors are related to the use of edible and medicinal plants; (c) analyze people perception discussing the importance of the culture and the environment for plant uses.

## Materials and methods

### Study Sites

The nine studied communities are located within the Chaco phytogeographic region [20].

The west area conforms the Chaco Arido (Dry Chaco) [20], characterized by the species with adaptations to drought, and scarce rainfalls (300 to 500 mm annual) mainly concentrated in the summer [21]. The original vegetation surrounding the studied communities comprises xerophilous forests dominated by *Aspidosperma quebracho-blanco *Schltdl. ("quebracho blanco"), *Prosopis *spp. ("algarrobos"), *Cercidium praecox *(Ruiz & Pav. Ex Hook) Harms ("brea"), *Ziziphus mistol *Griseb. ("mistol") and *Stetsonia coryne *(Salm-Dyck) Britton & Rose ("cardón") [20,22]. The diversity of these forests in the Chaco Árido is mainly modified by cattle ranch, wood exploitation and intentional fire.

The Chaco Serrano (Hill Chaco) extends along the three main mountain ranges of Cordoba (Sierras Chicas, Sierras Grandes, and Pocho-Guasapampa), from 400 to 1300 m a.s.l. [20]. This area is more humid than Chaco Árido, with greater plant richness, and presents a mosaic of generally small patches of forest communities intermingled with shrubs and grasslands. The remaining forests are dominated by *Lithraea molleoides *(Vell.) Engl. ("molle"), *Schinopsis marginata *Engl. ("orco quebracho"), Zanthonxylum coco Gilliet et Hook. f & Arn. ("coco"), *Condalia *spp. ("piquillín de la sierra") and *Ruprechtia apetala *Wedd. ("manzano del campo") [20]. The original vegetation has been drastically reduced due to deforestation and fire and has been replaced by large-scale agriculture fields or secondary forests of native and introduced species [23].

In the northern Chaco the slopes are lower, and the vegetation is characterized by the appearance of palms in highlands valleys and the existence of subtropical elements like "mato" *(Mycianthes cisplatensis*) [22]. Original forests have been modified dramatically by anthropic activities, as intentional fires, deforestation and cattle ranch. In general, a strong advance of the agriculture on the forest lands was observed during the last years [22].

We selected three biogeographical regions that offer some environmental differences among them (i.e., mean annual temperature, mean annual rainfall, etc.) as well as particularities in the composition of the flora: Chaco Serrano, Chaco Árido, and Northern Chaco with subtropical elements. The selection of these regions was made also considering potential socio-cultural differences between them, as NBI (Unsatisfied Basic Necessities, acronym in Spanish), tourism (simply if the community receive or not tourists frequently), and the isolation level of the community respect main important cities (i.e., more than 10,000 inhabitants).

**Figure 1:**
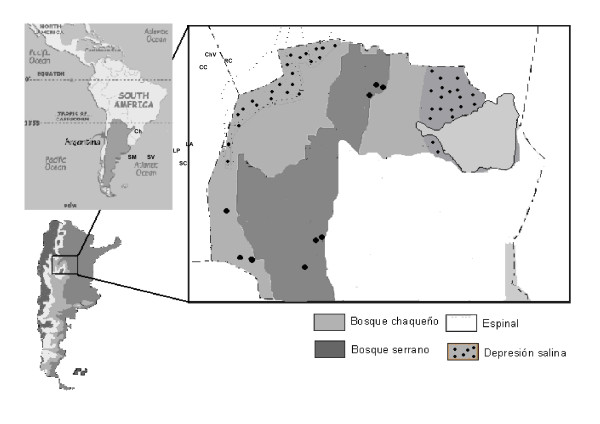
**Location of the studied communities within each of the three biogeographical regions of Córdoba province**. References: ChV: Chañar Viejo, RC: Rayo Cortado, CC: Cerro Colorado, LP: La Paisanita, LA: Los Aromos, SC: San Clemente, Ch: Chancaní, SM: San Martín, SV: San Vicente.

Within each of these biogeographical regions, we selected three rural communities that were considered as statistical replicates. Thus, a total of nine rural communities were sampled: *Chancaní, San Vicente *and *San Martín *within the Chaco Árido; *San Clemente, Los Aromos *and *La Paisanita *within the Chaco Serrano; and *Cerro Colorado, Rayo Cortado *and *Chañar Viejo *within the northern Chaco of Cordoba (Figure 1).

### Sampling Methods

We used available information on plant species richness and abundance for each region (unpublished inventories provided by M. Cabido and L. Galetto) to analyze phytogeographical differences, In addition, we performed new plant inventories for each of the communities located within the three studied regions.

Information on socio-cultural characteristics, and data on the knowledge and use of wild vegetables were collected through 192 interviews (one person per family), covering 80-100% of permanent residents' houses of each community. First we have explained to each family the aims of the interview, then the members designated the person to be interviewed. The total number of interviews were 32 in Chancaní, 28 in San Vicente, 13 in San Martín, 24 in San Clemente, 22 in Los Aromos, 12 in La Paisanita, 22 in Cerro Colorado, 25 in Rayo Cortado, and 14 in Chañar Viejo.

The interviews had two sections: one structured and the other semi-structured [24]. The first one was composed to obtain data on knowledge and use of the wild plant resources applying the free list method, and the second one to document the socioeconomic characterization (economic, geographic and educational variables). In addition, unstructured interviews about social role by gender, perceptions, preferences, collection and preparation modes of useful plants were performed. Finally, participant observations using voucher specimens to complete plant uses and to corroborate species recognition- [24] were carried out. The duration of the interviews ranged between 45 minutes to 2 hours.

All species were identified in the laboratory, and their scientific name was corroborated using the on-line version of the "Catálogo de Plantas Vasculares de la República Argentina" (Darwinion Institute, available on http://www.darwin.edu.ar, Febrary 2009). The voucher specimens will be deposited at the Herbarium of the Botany Museum (CORD, Universidad Nacional de Cordoba, Argentina).

### Statistical Analysis

Multiple regressions were carried out in order to explore the relationships between sociocultural variables and the uses of edible and medicinal plants.

Next, in order to explore the combined effects of variables related to the natural and cultural environments, a Principal Components Analysis (PCA - 25) was carried out. In this case, the main utility of PCA was to analyze the variability trying to detect some patterns. Overall variation was graphically represented in two dimensions, selecting those significant variables of each axis. These graphical tools help to identify some patterns in the data set. Many socio-cultural variables were considered for multiple regression and PCA analysis: gender, age (categorized as ≤ 45 and ≥ 46 years), job (stable, unstable), years of residence (categorized as 0-5, 6-10, 11-15, ≥ 16), NBI (the index varies from 0 in La Paisanita to 54 in Chancaní), and tourism (categorized as "present" or "absent").

In the PCA, the socio-cultural variables were considered in relation to the use of plants: average number of medicinal (or edible) plants know by men and by women (being each gender one variable) in each community, the same for age, job and years of residence (for example, the average number of medicinal plants know by persons with stable job is one variable, the average number of edible plants know by persons younger than 46 is another). Tourism was considered as a dummy variable.

Natural variation (i.e., differences in plant composition and availability) of plant resources was considered between three differential phytogeografical areas (Chaco Árido, Chaco Serrano, and Northern Chaco), sampling three communities in each of these areas. Information about plant knowledge was quantified by means of a "adjusted citation number" (a different value for each plant species) which corresponds to the number of citations for each species, divided by the number of surveys performed in each community. Thus, we obtained a comparative value for the knowledge of each plant species.

The resulting matrix was conformed by nine rows, one for each community and 126 columns, one for each average number of use (edible or medicinal plants) related to each level of each socio-cultural variable, one for NBI, one for "tourism", and one for the adjusted citation number of each plant.

Finally, all the variables were standardized before running the analysis.

For the graphic representation of PCA, species and socio-cultural variables with significant effects (i.e., obtained from the correlation between each variable and each axis) on the axis were selected. The scores ranged between 0 (i.e., in the absence of influence) and 1 (i.e., when the influence is maximum in the determination of the axis); we have considered only variables with scores higher than 0.5.

## Results

### General trends of plant uses for the Chaco region

A total of 21 edible and 120 medicinal plant species were registered in this study. It is interesting to emphasize that almost all known plants are used by people. By this reason the categories of known and used plants were homologated throughout this section, in order to simplify the presentation of the results.

The consumption of edible plants is mainly restricted to the occasional use of fruits of native species. The exceptions were "algarrobos" (*Prosopis *spp.), "tuna" (*Opuntia ficus-indica *(L.) Mill.) and "chañar" (*Geoffraea decorticans *(Gillies ex-Hook. & Arn.) Burkart), because these species are commonly used in all of the studied communities. Although these species are still frequently mentioned and used, people emphasize they were more commonly used in the past.

The properties of medicinal plants are appreciated in all the communities. Medicinal plants are mainly used to treat digestive disorders, and secondary as diuretics, febrifuge, and cough suppressant. Also, few species are occasionally used as disinfectant, analgesic, abortive, contraceptive, anti-inflammatory, depurative, sedative, and cardiotonic [See Table S1, additional file 1].

All diseases mentioned by rural people express popular concepts that may have not an equivalent in the western-scientific medicine. Particular details about the use of many plant species have been presented in previous contributions [26-28].

### Influences of cultural and environmental factors on the use of edible and medicinal plants

In a first step, for disentangle the importance of several sociocultural variables on the use of edible and medicinal plants a multiple regression was carried out. However, most of them (sex, age, job, years of residence, tourism, NBI) did not show significative relationships with the use of plants (results not showed). The exceptions was the variable tourism, which have slight influence on the use of edible plants (0.178, p = 0.014). The higher number of used edible plants is observed in communities with scarce tourism.

The results of the PCA showed that the percentage of the variance explained by the first two axes is of 48.7%. It is important to emphasize that the contribution of both axis is equivalent, since the first one explains 28.2% and the second one 20.5% of the total variance. Table 1 shows the contribution of each sociocultural variable used in the PCA. Table 2 shows the contribution of 30 species with significant influence in the resolution of the PCA analysis.

**Table 1:** Eigenvectors for the first two axis of each of the sociocultural variables used in the PCA

Sociocultural variables	C 1	C 2
NBI	0.103	-0.620

Tourism	-0.702	0.597

Food - Men	0.882	-0.096

Food - Women	0.798	0.357

Medicinal - Men	0.008	0.073

Medicinal - Women	-0.307	0.736

Food - <46	0.841	0.194

Food - > 45	0.941	0.180

Medicinal - <46	-0.577	0.223

Medicinal - >45	0.222	0.046

Food - Stable job	0.918	0.078

Food - Unstable job	0.845	0.386

Medicinal - Stable job	-0.151	0.730

Medicinal - Unstable job	-0.242	0.316

Food - 1a5 years of residence	0.531	-0.463

Food - 6a10 y o r	0.348	0.747

Food - 11a15 y o r	0.615	0.159

Food - 16 and+ y o r	0.798	0.359

Medicinal - 1a5 years of residence	0.176	-0.464

Medicinal - 6a10 y o r	-0.392	0.284

Medicinal - 11a15 y o r	0.080	-0.408

Medicinal - 16 and+ y o r	-0.016	0.760

**Table 2:** Eigenvectors for the first two axis of each of the species with significant influence in the resolution of the PCA analysis

	C 1	C 2
1. Peperina	-0.88	-0.1
2. Vira-vira	-0.88	-0.22
3. Doradilla	-0.86	-0.25
4. Cola de quirquincho	-0.72	-0.08
5. Suico	-0.74	-0.3
6. Canchalagua	-0.75	-0.41
7. Chañar	0.59	0.11
8. Mistol	0.62	0.03
9. Piquillín	0.85	-0.41
10. Algarrobo	0.88	0.19
11. Tuna	0.82	-0.14
12. Tusca	0.84	-0.07
13. Manzanilla	0.38	0.84
14. Menta	0.05	0.85
15. Albahaca	0.26	0.82
16. Cepa caballo	0.47	0.77
17. Tilo	0.1	0.73
18. Poleo del campo	0.23	0.7
19. Ruda	0.29	0.68
20. Pulmonaria	-0.26	-0.84
21. Pan de cata	0.47	-0.86
22. Duraznillo	0.37	-0.8
23. Uvita del campo	0.17	-0.78
24. Cola de caballo	-0.52	-0.72
25. Quimpe	0.29	-0.75
26. Tomillo	0.38	-0.74
27. Quebracho flojo	0.62	-0.73
28. Tunilla	0.67	-0.68
29. Ucle	0.67	-0.68
30. Uluba	0.67	-0.68

**Figure 2:**
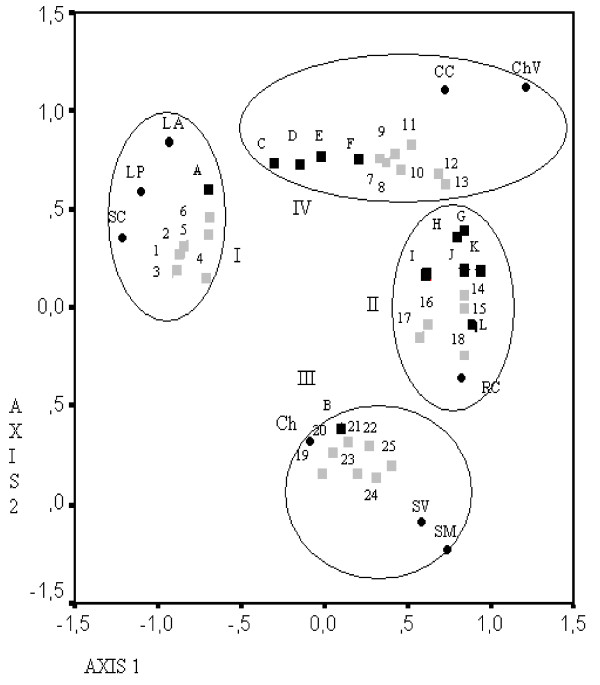
**PCA showing the distribution of useful plant species, socio-cultural variables and the nine communities from three different biogeographic regions of Córdoba, Argentina**. Abbreviations: SC: San Clemente, LP: La Paisanita, LA: Los Aromos, CC: Cerro Colorado, ChV: Chañar Viejo, RC: Rayo Cortado, CH: Chancaní, SV: San Vicente, SM: San Martín. A: tourism, B: NBI, C: medicinal plants/women, D: med.plants/stable work, E: medicinal plants/16&+years of residence, F: food plants/6 to10 years of residence, G: food plants/unstable work, H: food plants/16&+ years of residence, I: food plants/women, J: food plants/under 45 years old, K: food plants/46&+years old, L: food plants/men (/: used by). 1: vira.vira, 2: doradilla, 3: peperina, 4: cola de quirquincho, 5: suico, 6: canchalagua, 7: uvita, 8: quimpe, 9: duraznillo, 10: tomillo, 11: pan de cata, 12: quebracho flojo, 13: ucle, 14: tuna, 15: tusca, 16: mistol, 17: chañar, 18: algarrobo, 19: tilo, 20: poleo, 21: ruda, 22: menta, 23: albahaca, 24: cepa-caballo, 25: manzanilla (scientific names in text and in Table 1). Black circle - Communities Grey square - Species Black square - Socio-cultural factors.

Figure 2 shows the distribution of the nine communities from three different biogeographic regions and the most significant socio-cultural variables and plant species that contributed in the resolution of the PCA analysis.

Four groups (circles) can be visualized on Figure 2.

**Group I **comprises the communities of Chaco Serrano (Los Aromos, San Clemente and La Paisanita) along with species of higher abundance and uses in that region - species 1 to 5 in Table 3, as "doradilla" (*Anemia tomentosa *(Savigny) Sw.), "viva-vira" (*Achyrocline satureioides *(Lam.) DC.), "suico" (*Tagetes minuta *L.), "peperina" (*Minthostachys mollis *(Kunth.) Griseb.), "cola de quirquincho" (*Huperzia saururus *(Lam.) Trevis)-, in addition to the presence of tourism.

Group II comprehends the community of Rayo Cortado (northern Chaco), species of trees with big and sweet fruits - "mistol" (*Ziziphus mistol *Griseb.), "chañar" (*Geoffraea decorticans *(Gillies ex Hook. & Arn.) Burkart), "tusca" (*Acacia aroma *Gillies ex Hook & Arn.), "tuna" (*Opuntia ficus-indica *(L.) Mill.), "algarrobos" (*Prosopis *spp.), 7 to 12 in Table 3-, and socio-cultural variables (gender, age, time of residence and job type) correlated with the consumption of edible plants.

Group III includes the Chaco Árido communities-Chancaní, San Vicente and San Martín-, in addition to many medicinal plants frequently used there, species 13 to 19 in table 3, such as "menta" (*Mentha *spp.), "tilo" (*Tilia *spp.), "albahaca" (*Ocimum basilicum *L.), "ruda" (*Ruta chalepensis *L.), "poleo del campo" (*Lippia turbinata *Griseb.), and "cepacaballo" (*Xanthium spinosum *L.). Most of them are exotic and habitually cultivated in gardens or yards. The NBI factor is related to this group.

The two remaining communities of northern Chaco (Cerro Colorado and Chañar Viejo) are within Group IV, together with some native medicinal species exclusively or mainly used there (20 to 30 in table 3, as "quebracho flojo" (*Jodinia rhombifolia *(Hook. et Arn.) Reissex), "tunilla" (*Opuntia sulphurea *Gillies ex Salm-Dyck), "quimpe" (*Coronopus didymus *(L.) Sm.), "tomillo" (*Thymus vulgaris *L), "pan de cata" (*Porlieria microphylla *(Baill.) Descole, O'Donell & Lourteig), "uvita" (*Salpichroa origanifolia *(Lam.) Baill.), "ucle" (*Cereus forbesii *Otto ex C.F.Först var. pampeana (Speg.) Backeb.), "uluba" (*Harrisia pomanensis *(F.A.C.Weber ex K. Schum.) Britton & Rose var. pomarensis), and some socio-cultural variables (gender: feminine, job type, and period of residence) that can be related to the use of plants.

## Discussion and conclusion

A high number of medicinal plants are currently used by rural people. In contrast, a scarce use of edible plants was registered. Nevertheless, complex relationships involving socio-cultural variables, a diversity of plant uses, and regional communities were evidenced.

The scarce use of edible plants can be related to general cultural patterns of Argentinean people. Aguirre [29] pointed out for the Argentineans, in general, the basic food is "meat with something else", except for people with high incomes of big cities, In addition, in Argentina as well as in other Latin American populations, wild food consumption is interpreted by many people as a sign of poverty [29,30]. Thus, if native resources are not considered "true" and valuable food by the inhabitants of rural communities of Cordoba, knowledge on edible native plants will be quickly eroded from the entire region. The exceptions are some native fruits occasionally consumed mainly by children or men during walks or horse rides, but these plant resources do not replace food usually bought in markets.

The widespread consumption of medicinal plants can be related to some particularities of the ethno-medical system used by rural people from Córdoba [26]. The high number of plant used as well as the diversity of well-known recipes, are evidences of the importance of medicinal plants for rural societies.

The results of the PCA comprehend three different criteria by grouping communities and socio-cultural variables. First, communities of the Chaco Serrano forest showed a general pattern (i.e., the use of species with higher abundance within that region) indicating a prevalent environmental influence. Second, communities of the Chaco Árido forest are grouped mainly by NBI and the use of a particular set of exotic and cultivated plants with a high cultural valuation, indicating a prevalent socio-cultural influence. Third, communities of the northern Chaco (groups II and IV) are grouped by a combination of socio-cultural variables with several edible and medicinal plants. However, the species most used by these two groups are not the more abundant in this phytogeographical region, suggesting the existence of additional cultural factors influencing the decision on plant uses.

Thus, even when the influence of the natural environment on the selection of useful plants can not be underestimated, culture seems to play a more important role on that selection. Among the socio-cultural factors, gender (feminine) can be related with the use of medicinal plants, aspect on which the settlers were consulted. Interviewed persons explained that the maintenance of gardens of medicinal plants, familiar health care, and the administration of therapies, are women responsibilities. The relationship between gender and the use of plants has been pointed out previously for other regions (e.g. [14,31,32]), as well as for the Chaco of Córdoba [26-28].

Moreover, the association of plants from communities of the Chaco Árido forest with exotic highly valued species [33], e.g., "ruda", *Ruta chalepensis *L., mentioned as "protective of home", and still widely used at the Mediterranean region [34], is a signal of the existence of intrinsic cultural rules into these populations. In fact, those exotic species must be cultivated and protected to prosper, even when native plants growing in the area with similar properties in the traditional medicine are available.

The use of exotic species has been mentioned in the bibliography [35] as indicative of the privileged effect of culture on environmental availability about the use of medicinal plants. In this case, most valuation of the curative power attributed to these species comes from old traditions with Mediterranean origin brought to the Americas by the Spaniards [33]. For example, the case of the "ruda" is particular by the ample diffusion of the belief about its supernatural powers.

Although references only provide few recent data on plant uses with this integrated view, some coincidences can be pointed out. One of these studies [35], analyzed the influence of ecological factors (i.e. the distribution of the species of plants) and cultural variables (i.e., traditions) on the use of 18 medicinal species in Navarre, concluding that the cultural factors are decisive in the selection of these plants. Weckerle et al. [36], studying the knowledge on plant uses in mountainous areas of China, concluded that culture seems to have greater influence than the environment. Indeed, our results from a large biogeographical scale showed that the use of plants is part of a very complex system involving natural and socio-cultural variables. Thus, in our opinion, ethnobiological studies trying to describe and understand the relationships between social systems and the environment should include all the possible dimensions within the analysis.

On the other hand, we consider that our methodological approach (i.e., using species composition by means of the "citation index" instead of number of mentioned plants as usually, as well as the PCA) may be useful for the discipline since it allows to easily visualize the associations between multiple factors, and to analyze the complex system conformed by people, nature and culture. In addition, a better understanding of ecological complexity and rural knowledge and management contributes to develop effective conservation policies [37]

In conclusion, this study showed that medicinal species are widely used, whereas the knowledge on edible plants is disappearing. Both tendencies can be related to different cultural rules of valuations. In that sense, the comprehensive approach to study different variables related to the environment and the culture in the use of plants at a regional scale shows how complex the system can be. In addition and considering the conservation point of view, these results alert about the importance of re-valuing the natural resources taking into account socio-cultural meanings, mainly in areas suffering intense deforestation. Our data evidenced that, even when plant natural availability (i.e., the environment) is always important, the cultural environment has the strong influence on the use of medicinal and edible plants in rural communities of the Chaco of Córdoba (Argentina).

## Competing interests

The authors declare that they have no competing interests.

## Authors' contributions

All authors participated in the planning and design of the work. BAT carried out the field work, performed statistical analyses and made interpretation of data. LG and SC supervised the research works and the statistical analysis. BAT wrote a first version of the manuscript, LG and SC revised the manuscript; all authors read and approved the final manuscript.

## Supplementary Material

Additional file 1 **Table S1**. Medicinal (120 spp.) and edible (21 spp.) plants used in the studied communities. Family, scientific and common names, origin and use are included. It provides a complete list of edible and medicinal species, including their scientific and vernacular names, origin, organs of the plant used, and disease or disorder treated. Click here for file
