# Supplementary material for: Ethnobotanical knowledge in rural communities of Cordoba (Argentina): the importance of cultural and biogeographical factors
Source: J Ethnobiol Ethnomed. 2009 Dec 15;5:40. doi: 10.1186/1746-4269-5-40 (PMC2804588; doi:10.1186/1746-4269-5-40)
Supplement: Additional file 1 — Table S1. Medicinal (120 spp.) and edible (21 spp.) plants used in the studied communities. Family, scientific and common names, origin and use are included. It provides a complete list of edible and medicinal species, including their scientific and vernacular names, origin, organs of the plant used, and disease or disorder treated. [file 1746-4269-5-40-S1.PDF]

Table 1. Medicinal (120 spp.) and edible (21 spp.) plants used in the studied communities

MEDICINAL PLANTS				
**FAMILY**	**SCIENTIFIC NAME**	**COMMON NAME**	**ORIGIN**	**USE**
**Amaranthaceae**	*Alternanthera pungens* Kunth	Yerba del pollo	N	Digestive disorders, diuretic, diarrhea, "hot diarrhea" (caused by sun), "empacho"
**Anacardiaceae**	*Lithraea molleoides (Vell.) Engl.*	Molle	N	Tooth pain, sedative, digestive
	Schinus areira L.	Aguaribay	N	Digestive disorders, abortive, head ache, anti-inflammatory, healing.
	Schinus fasciculata (Griseb) I.M. Johnst.	Moradillo	N	Circulation, tooth pain
**Apiaceae**	*Foeniculum vulgare Mill..*	Hinojo	Natur.	……
	Petroselium crispum (Mill.) A.W. Hill	Perejil	Intr.	Abortive
**Apocynaceae**	*Aspidosperma quebracho-blanco* Schltdl.	Quebracho blanco	N	Disinfectant, digestive disorders, contraceptive
**Aristolochiaceae**	*Aristolochia argentina* Griseb.	Charruga	N	Diuretic, diarrhea
**Asclepiadaceae**	*Morrenia odorata* (Hook. & Arn.) Lindl.	Tasi	N	Skin disorders
**Asteraceae**	*Achyrocline satureioides* (Lam.) DC.	Vira-vira	N	Digestive disorders, respiratory disorders
	Achyrocline tomentosa Rusby	Marcela	N	Digestive disorders, diarrhea
	Artemisia annua L.	Ajenjo - Fernet	Natur.	Digestive disorders
	Artemisia douglasiana Besser	Matico - Hepatalgina - Buscapina	Natur.	Digestive disorders, hepatic disease
	Baccharis articulata (Lam.) Pers.	Carquejilla	N	Hepatic disease
	Baccharis crispa Spreng.	Carqueja	N	Hepatic disease
	Bidens subalternans DC.	Manzanilla silvestre	N	Digestive disorders
	Conyza sp.	Pulmonaria	N	Respiratory disorders
	Cyclolepis genistoides D. Don	Palo azul	N	Back ache
	Jungia polita Griseb.	Zarzaparrilla	N	Diuretic, circulation, to fall the blood pressure
	Lactuca sativa	Lechuga	Natur	Sedative

	L.			
	Matricaria recutita L.	Manzanilla	Natur	Digestive disorders, disinfectant, antibiotic, anti-inflammatory
	Schkuhria pinnata (Lam.) Kuntze ex Thell.	Canchalagua	N	Digestive disorders, hepatic disease
	Tagetes filifolia Lag.	Anisillo	N	…
	Tagetes minuta L.	Suico	N	Parasits, diarrhea, digestive disorders
	Taraxacum officinale Weber ex F.H. Wigg.	Diente de león	Natur.	Digestive.
	Tessaria dodoneifolia (Hook. et Arn.) Cabrera	Suncho	N	
	Trixis divaricata (Kunth) Spreng.	Contrayerba	N	Digestive disorders, knocks, pains
	Xanthium spinosum L.	Cepa caballo	N	Back ache, to blood
**Bignoniaceae**	*Dolichandra cynanchoides* Cham.	Mil hombres	N	…
**Boraginaceae**	*Borago officinalis* L.	Borraja	Natur.	Cough suppressant
**Brassicaceae**	*Coronopus didymus* (L.) Sm.	Quimpe	N	To cold/flu, to blood, diuretic, cough suppressant
	Lepidium bonariense L.	Bolsa de pastor	N	Diarrhea
**Buddlejaceae**	*Buddleja cordobensis* Griseb.	Salvialora	N	Respiratory disorders, “aire”
**Cappareceae**	*Capparis atamisquea* Kuntze	Atamisqui	N	Disinfectant, tooth pain, “pasmo de sol”
**Cecropiaceae**	*Cecropia pachystachya* Trécul	Ambay	N	Cough suppressant
**Celtidaceae**	*Celtis ehrenbergiana* (Klotzsch) Liebm.	Tala	N	Children’s digestive disorders, “aire”
**Commelinaceae**	*Commelina erecta* L.	Santa Lucía	N	To eyes
**Chenopodiaceae**	*Atriplex undulata* (Moq.) D. Dietr	Cachiyuyo	N	…
	Chenopodium album L.	Quina	Intr.	…
	Chenopodium ambrosioides L.	Paico	N	Digestive disorders
**Ephedraceae**	*Ephedra triandra* Tul. emend. J.H. Hunz	Tramontana	N	To knocks, back aches
**Equisetaceae**	*Equisetum giganteum* L.	Cola de caballo	N	Back aches, diuretic

**Euphorbiaceae**	*Acalypha communis* Müll. Arg.	Albaquilla	N	Digestive disorders
	Croton sarcopetalus Müll. Arg.	Cambalacho	N	Depurative, skin disorders
	Euphorbia serpens Kunth	Yerba meona	N	Diuretic, to flu
	Ricinus communis L.	Castor	Natur.	Laxative
**Fabaceae**	*Acacia aroma* Gillies ex Hook & Arn.	Tusca	N	Disinfectant, to knocks, back aches
	Acacia caven (Mol.) Mol.	Espinillo - Churqui	N	Disinfectant, antibiotic, tooth pain
	Acacia praecox Griseb.	Garabato	N	…
	Bauhinia forficata Link subsp. *pruinosa* (Vog.) Fortunato et Wonderlin	Pezuña de vaca	N	Diabetes
	Caeselpinia gilliesii (Wall. ex Hook.) D. Dietr.	Lagaña de perro	N	Tooth pain
	Geoffraea decorticans (Gillies ex Hook. & Arn.) Burkart	Chañar	N	Respiratory disorders
	Prosopis alba Griseb.	Algarrobo blanco	N	Diarrhea
	Senna sp.	Sen	Natur.	Digestive disorders
**Gentianaceae**	*Gentianella* spp.	Genciana	Natur.	…
**Hydnoraceae**	*Prosopanche americana* (R.Br.) Baillon	Guaycurú - Flor de piedra	N	Respiratory disorders, hepatic disease
**Hypericaceae**	*Hypericum connatum* Lam.	Cabo torilo	N	Heart disease
**Lamiaceae**	*Hedeoma multiflora* Benth.	Tomillo	Intr.	Digestive disorders, disinfectant, head ache
	Lavandula sp.	Lavanda	Intr.	Sedative
	Lepechinia floribunda (Benth.) Epling	Salvia blanca	N	Respiratory disorders
	Marrubium vulgare L.	Yerba del sapo - Marrubio - Malva amarga	Natur.	Hepatic disease
	Melissa officinalis L.	Toronjil-Melisa	Natur.	Heart disease
	Mentha sp.	Menta	Intr.	Digestive disorders, anti-inflammatory, period pain
	Mentha sp.	Veramota	Intr.	Digestive disorders
	Mentha sp.	Yerbabuena	Intr.	Digestive disorders, to flu, diarrhea
	Minthostachys mollis (Kunth.) Griseb.	Peperina	N	Digestive disorders, to fall the blood pressure, abortive
	Ocimum basilicum L.	Albahaca	Intr.	Digestive disorders, “pasmo”
	Origanum sp.	Orégano	Intr.	Digestive disorders

	Plectranthus madagascariense (Pers.) Benth.	Incienso	E	To perfume
	Rosmarinus officinalis L.	Romero	Intr.	Digestive disorders, hepatic disease, to flu, to eyes
	Salvia officinalis L.	Salvia	Intr.	Digestive disorders, sedative
**Lamoriopsidaceae**	*Elaphoglossum gayanum* (Feé) T. Moore	Calaguala		Period pain
**Lauraceae**	*Cinnamomum* sp.	Alcanfor	Intr.	"Aire", anti-parasite, repellent
	Laurus nobilis L.	Laurel	Intr.	Digestive disorders, respiratory disorders
**Liliaceae**	*Aloe* spp.	Aloe Vera	E	Skin disorders
**Loranthaceae**	*Ligaria cuneifolia* (Ruiz et. Pav) Tiegh.	Liga	N	To regulate the blood pressure
**Lycopodiaceae**	*Huperzia saururus* (Lam.) Trevis	Cola de quirquincho	N	Aphrodisiac
**Malvaceae**	*Malvastrum coromandelianum* (L.) Garcke	Yerba del potro	N	Hepatic disease
	Sphaeralcea bonariensis (Cav.) Griseb.	Malvarisco	N	Slow intestinal activity
	Sphaeralcea cordobensis Krapov.	Malva - Malva dulce	N	Injuries, conjunctivitis, period pain, back aches, digestive disorders
**Meliaceae**	*Melia azedarach* L.	Paraíso	Natur.	…
**Myrtaceae**	*Eucalyptus* spp.	Eucaliptus	E	Respiratory disorders
**Olacaceae**	*Ximena americana* L.	Albarillo	N	…
**Oleaceae**	*Fraxinus* sp.	Fresno	E	…
**Passifloraceae**	*Passiflora caerulea* L.	Pasionaria	N	Sedative, diarrhea, respiratory disorders
**Phytolaccaceae**	*Phytolacca dioica* L.	Ombú	N	Laxative
**Plantaginaceae**	*Plantago major* L.	Llantén	Intr.	Digestive disorders, hepatic disease, back aches, disinfectant, etc
**Poaceae**	*Tritucum* sp., *Paspalum* sp.	Gramilla	N	Back aches, abortive, contraceptive
**Polygonaceae**	*Rumex crispus* L.	Lengua de Vaca	Natur.	…
	Polygonum sp.	Sanguinaria	Natur.	Blood circulation
**Ramnaceae**	*Condalia buxifolia* Reissek	Piquillín	N	…
**Ranunculaceae**	*Clematis montevidensis* Spreng.	Loconte	N	…
**Rhamnaceae**	*Ziziphus mistol* Griseb.	Mistol	N	Respiratory disorders
**Rosaceae**	*Prunus persica* (L) Batsch	Durazno (hoja)	Intr.	Digestive disorders, respiratory disorders
	Eriobotrya japonica (Thunb.) Lindl.	Níspero	E	Respiratory disorders
**Rutaceae**	*Citrus limon*	Limón	Intr.	Digestive disorders, respiratory disorders

	(L.) Burm.			
	Citrus sinensis (L.) Osbeck	Naranjo (hoja)	Intr.	Sedative, cough suppressant
	Ruta chalepensis L.	Ruda	Intr.	Digestive disorders, home protector, blood circulation, sedative
**Salicaceae**	*Salix* sp.	Sauce	…	Analgesic
**Santalaceae**	*Jodinia rhombifolia* (Hook. et Arn.) Reissex	Quebracho flojo - Sombra de toro	N	Digestive disorders, respiratory disorders, diarrhea, abortive, etc
**Schizaeaceae**	*Anemia tomentosa* (Savigny) Sw.	Doradilla	N	Period problems, abortive, back aches
**Solanaceae**	*Cestrum parqui* L´Hér.	Duraznillo negro	N	…
	Lycium cestroides Schltdl.	Ruminico	N	…
	Nicotiana glauca Graham	Palán-palán	N	Skin diseases, injuries
	Solanum argentinium Bitter & Lillo	Duraznillo	N	To sunstroke, tooth pain, “pasmo”
	Solanum sisymbriifolium Lam.	Espina colorada	N	Back ache, sore throat, digestive disorders
**Tiliaceae**	*Tilia* sp.	Tilo	E	Sedative
**Urticaceae**	*Urtica urens* L.	Ortiga	Natur.	Blood circulation, back aches, cough suppressant, hair loss
**Usneaceae**	*Usnea hieronymi* Krempelh	Barba de piedra	N	Sore throat
**Valerianaceae**	*Valeriana* sp.	Valeriana	E	Sedative
**Verbenaceae**	*Aloysia citriodora* Ortega ex Pers.	Cedrón	N	Heart diseases, digestive disorders
	Aloysia gratissima (Gillies & Hook. ex Hook.) Tronc.	Palo amarillo	N	Digestive disorders, “levantar el ánimo” (to cheer up), “enfriamiento”
	Aloysia polystachya (Griseb.) Moldenke	Poleo de burro (Té de)	N	Digestive disorders
	Glandularia dissecta (Willd. ex Spreng.) Schnack & Covas	Verbena	N	Sedative
	Lippia integrifolia (Griseb.) Hieron.	Incayuyo	N	Digestive disorders, back ache
	Lippia turbinata Griseb.	Poleo del campo	N	Digestive disorders, “levantar el ánimo” (to cheer up), “enfriamiento”
**Zygophyllaceae**	*Larrea divaricata* Cav.	Jarilla	N	To bath, skin diseases, to knocks
**Zygophyllaceae**	*Porlieria microphylla* (Baill.) Descole, O´Donell & Lourteig	Pan de cata - Cucharero - Guayacán	N	Hepatic disease, cough suppressant, diarrhea

**...**	NI	Marquito	...	...
**...**	NI.	Mimosa	...	Heart diseases

EDIBLE PLANTS				
**FAMILY**	**SCIENTIFIC NAME**	**COMMON NAME**	**ORIGIN**	**USED PART**
**Anacardiaceae**	*Lithraea molleoides* (Vell.) Engl.	Molle	N	Fruit
**Asteraceae**	*Carduus* sp.	Cardo	Adv.	Leaves
	Taraxacum officinale Weber ex F.H. Wigg.	Diente de león	Adv.	Leaves
**Boletaceae**	*Boletus luteus* L. o *Suillus luteus* (L. ex Fr.) S. F. Gray	Hongos del pino	N	Complete plant
**Cactaceae**	*Opuntia ficus-indica* (L.) Mill.	Tuna	Intr.	Complete plant
	Opuntia sulphurea Gillies ex Salm-Dyck	Tunilla - Tuna colorada	N	Fruit
	Cereus forbesii Otto ex C.F.Först var. pampeana (Speg.) Backeb.	Ucle	N	Fruit
	Harrisia pomanensis (F.A.C.Weber ex K. Schum.) Britton & Rose var. pomarensis	Uluba	N	Fruit
**Celtidaceae**	*Celtis ehrenbergiana* (Klotzsch) Liebm.	Tala	N	Fruit
**Fabaceae**	*Prosopis* spp.	Algarrobo	N	Fruit
	Geoffraea decorticans (Gillies ex Hook. & Arn.) Burkart	Chañar	N	Fruit
**Gyrodontaceae**	*Phlebopus bruchii* (Speg.) Heinem. & Rammeloo	Hongos del coco	N	Complete plant
**Moraceae**	*Morus alba* L.	Mora	Adv.	Fruit
**Olacaceae**	*Ximena americana* L.	Albarillo	N	Fruit
**Passifloraceae**	*Passiflora caerulea* L.	Pasionaria	N	Fruit
**Rhamnaceae**	*Condalia buxifolia* Reissek	Piquillín	N	Fruit
	Ziziphus mistol Griseb.	Mistol	N	Fruit
**Rosaceae**	*Duchesnea indica* (Andrews) Focke	Frutillita del campo	Natur.	Fruit
**Smilaceae**	*Smilax campestris* Griseb.	Zarzamora	...	Fruit
**Solanaceae**	*Salpichroa origanifolia* (Lam.) Baill.	Uvita del campo	N	Fruit
**...**	NI	Berro	...	Leaves

Abbreviations: N: native plants, E: exotic plants, Intr.: introduced plants, Natur.: naturalised plants, NI: no identified material, ...: no data found.
“Aire” and “Pasmo” are popular diseases without correlation in the scientific medical system, related to the imbalance between internal and external temperatures; “Empacho” also is a popular disease equivalent to a indigestion.
